# Innovative Strategies in X-ray Crystallography for Exploring Structural Dynamics and Reaction Mechanisms in Metabolic Disorders

**DOI:** 10.3390/jpm14090909

**Published:** 2024-08-27

**Authors:** Alice Grieco, Isabel Quereda-Moraleda, Jose Manuel Martin-Garcia

**Affiliations:** Department of Crystallography and Structural Biology, Institute of Physical Chemistry Blas Cabrera, Spanish National Research Council (CSIC), 28006 Madrid, Spain; agrieco@iqf.csic.es (A.G.); iquereda@iqf.csic.es (I.Q.-M.)

**Keywords:** metabolic disorders, protein dynamics, enzyme catalysis, X-ray crystallography, microcrystals, time-resolved serial crystallography, XFELs

## Abstract

Enzymes are crucial in metabolic processes, and their dysfunction can lead to severe metabolic disorders. Structural biology, particularly X-ray crystallography, has advanced our understanding of these diseases by providing 3D structures of pathological enzymes. However, traditional X-ray crystallography faces limitations, such as difficulties in obtaining suitable protein crystals and studying protein dynamics. X-ray free-electron lasers (XFELs) have revolutionized this field with their bright and brief X-ray pulses, providing high-resolution structures of radiation-sensitive and hard-to-crystallize proteins. XFELs also enable the study of protein dynamics through room temperature structures and time-resolved serial femtosecond crystallography, offering comprehensive insights into the molecular mechanisms of metabolic diseases. Understanding these dynamics is vital for developing effective therapies. This review highlights the contributions of protein dynamics studies using XFELs and synchrotrons to metabolic disorder research and their application in designing better therapies. It also discusses G protein-coupled receptors (GPCRs), which, though not enzymes, play key roles in regulating physiological systems and are implicated in many metabolic disorders.

## 1. The Role of Enzyme Dynamics in Metabolic Diseases

Enzymes are essential proteins that catalyze biochemical reactions, driving vital processes such as energy production, molecule synthesis, and waste removal within cells [1]. By lowering the energy required for reactions, enzymes ensure that metabolic processes occur rapidly enough to sustain life. For instance, orotidine 5’-phosphate decarboxylase speeds up a reaction that would otherwise take millions of years to milliseconds [2].

In human health, balanced enzyme activity is crucial for maintaining metabolic homeostasis. Disruptions to this balance can lead to metabolic diseases, including cardiovascular and neurological disorders, diabetes, and obesity, affecting millions globally. Metabolic syndrome—a cluster of conditions like obesity, high blood pressure, and impaired glucose levels—significantly increases the risk of heart disease, stroke, and type 2 diabetes [3,4]. Furthermore, metabolic enzyme dysregulation is linked to cancer progression and drug resistance [5]. Understanding these enzymes’ roles in disease is key to developing new treatments. As metabolic disorders rise globally, advancing research into enzyme function is critical for developing innovative diagnostic tools and therapies [6,7].

**Figure 1:**
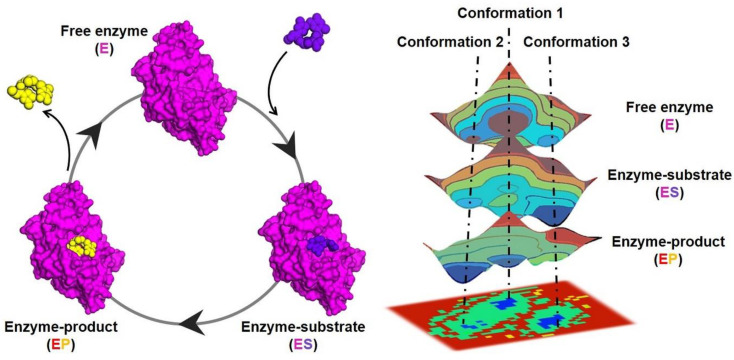
Schematic representation of a catalytic cycle (left) of a model enzyme (magenta) and the associated conformational changes represented in the free-energy landscape (right) at the different steps: free enzyme (E), enzyme-substrate (ES), and enzyme-product (EP). The energy distribution is shown by the coloring pattern: blue defines the conformational space with minimum energy (stable state), while brown defines a conformational space with maximum energy (unstable state). Transient local energy states are defined by intermediate color patterns. The substrate and the product of the reaction are shown as balls represented in purple and yellow, respectively.

Structural biology plays a pivotal role in identifying metabolic enzymes as therapeutic targets and understanding their role in disease. However, life at the molecular level is fundamentally dynamic. Despite decades of research studying and dissecting natural enzymes, a fundamental question that remains is how structural fluctuations (i.e., dynamics) in enzymes facilitate the efficient catalysis of complex multistep chemical transformations. Answering this fundamental question will have profound impacts on biology and biotechnology by not only allowing us to understand how enzymes are able to achieve their large rate enhancements but also by helping us gain insight into the evolution of novel enzyme activities and create efficient artificial biocatalysts from scratch for therapeutic and industrial applications. The 3D structures of enzymes indeed provide very relevant information on the arrangement of the catalytic machinery and structural elements composing the active-site pocket, but understanding enzymatic function requires the exploration of the ensemble of thermally accessible conformations that enzymes adopt in solution. Enzymes are flexible macromolecules that sample multiple conformational states, which are described by an energy landscape (Figure 1). Each conformational state in the ensemble has a specific distribution profile that can be portrayed by a free-energy landscape; lower-energy conformations are sampled (or populated) more frequently than those of higher energy [8,9]. The ability to shift between conformational substates is essential for their function, as they must undergo structural rearrangements during their catalytic cycle to promote substrate binding, stabilize transition state(s) and other high-energy reaction intermediates, and release product. Conformational changes that can directly impact catalytic function include side-chain conformational changes on fast timescales (picoseconds to nanoseconds), loop motions often playing a key role in substrate binding/product release on slower timescales (microseconds to milliseconds), and the allosteric transitions observed in some enzymes usually correspond to the slowest processes (milliseconds to seconds). Thus, understanding the basis of life and its evolutionary diversity requires deciphering not only the 3D structure but also the dynamic nature of enzymes (underlying living processes) and how they ensemble and function in a coordinated, timely and precise manner. Addressing this challenge will allow us to understand and treat diseases, harness biological processes for biotechnological purposes and synthetically design new enzymes.

In this review, we embark on a journey into the fascinating world of enzymes and their pivotal role in metabolic diseases. Through this exploration, we aim to shed light on the intricate molecular pathways that govern human health and offer insights into potential avenues for disease prevention, diagnosis, and treatment.

## 2. Structural Biology Approaches to the Study of Enzyme Dynamics in Metabolic Diseases

Since its beginning, structural biology research has made enormous strides in the study of fundamental biological processes and made a substantial contribution to our knowledge of the molecular mechanisms behind human diseases at an atomic resolution [10]. All biochemical reactions within cells are catalyzed by enzymes. Hence, they are the sustenance of life, and this makes them a valuable target in scientific research. The behavior of enzymes, i.e., catalysis, impacts the correct functioning of living beings. Several approaches have helped advance our understanding of enzyme dynamics and catalysis. On the one hand, experimental methods can be used to assess enzyme behavior: (i) fluorescence and Förster resonance energy transfer (FRET) can monitor enzyme dynamics and conformational changes in real time but requires labeling, which may perturb the system and alter the native conditions; and (ii) structural biology techniques provide high-resolution 3D structures of enzymes (X-ray crystallography), can probe enzyme dynamics and conformational changes in solution (nuclear magnetic resonance spectroscopy), and allow visualization of large enzyme complexes and conformational states (cryo-electron microscopy). However, all of them struggle to capture the full range of motions and timescales involved and are also limited by the protein size [11]. On the other hand, computational techniques can also be applied in the study of enzymes, as in the case of (i) molecular dynamics simulations, which model enzyme dynamics and conformational changes at an atomic resolution but are limited by accessible timescales and force field accuracy, and (ii) molecular mechanics, which accurately model enzyme-catalyzed chemical reactions but are computationally expensive. In general, computational approaches are hampered by the accuracy and system size [12]. Thanks to all these techniques, the field of structural biology has come a long way since the first crystal structure of a protein, the myoglobin from a sperm whale, was solved in 1958 [13]. As of August 2024, the Protein Data Bank repository has more than 224,000 entries of structural data determined from X-ray crystallography, nuclear magnetic resonance as well as electron microscopy and more than 1,000,000 computer structure models by AlphaFold2 (https://www.rcsb.org/stats/all-released-structures, accessed on 15 August 2024).

Recent advancements in protein structure determination have revolutionized our understanding of multi-component protein complexes. Innovative technologies, including automated chromatography platforms for purification and optimized crystallization strategies, have enabled the comprehensive characterization of proteins. By integrating various techniques, such as size exclusion chromatography, analytical ultracentrifugation, spectroscopy, and kinetics analysis, researchers can derive crucial insights into oligomeric assembly, conformational changes, enzyme catalysis, and ligand-protein interactions. There is no doubt that structural biology has played a crucial role in determining the molecular roles and mechanisms of various target proteins, such as the hundreds of metabolic enzymes linked to inborn errors of metabolism in humans [14,15]. Enzymes involved in inborn metabolic disorders are perfect candidates to be studied by structural biology because of two pivotal characteristics. (1) Most mutations that cause the disease are found in the exonic region of the mutated gene, not in the non-coding introns. These mutations generally affect the functionality and the catalysis of the enzyme as well as its overall globular shape, making these structures suitable to be determined by structural biology. (2) More than 65% of these mutations are missense type mutations, which alter just a single amino acid. Most pathogenic missense mutations cause a specific molecular phenotype through protein destabilization [16]. The structural biology field is currently advancing toward a high-resolution image of the cell, evaluating metabolic networks and the linkage with cancer patterns at ever higher levels of complexity. It seems obvious that these paths will lead to an ever more comprehensive understanding of the molecular pathways behind human metabolic disorders and cancer progression. With this aim in mind, among the structural biology techniques, X-ray crystallography has important advantages, such as the atomic and time resolution.

## 3. X-ray Crystallography and Its Role in Enzyme Dynamics

X-ray crystallography stands out among all these approaches as the technique that has provided the largest number of high-resolution protein structures, both in their apo-form and bound to their ligands [17]. Specifically, X-ray crystallography determines the atomic and molecular structure of biomolecules’ and inorganic compounds’ crystals. This method has been fundamental in understanding the structure and function of biological molecules, including enzymes, by providing detailed insights into their molecular architecture and the arrangement of atoms within them. In enzyme research, X-ray crystallography has provided key structural insights into the molecular basis of enzymatic reactions. By determining the atomic structure of enzymes, X-ray crystallography helps in characterizing their active sites, understanding their catalytic mechanisms, and facilitating structure-based drug design, site-directed mutagenesis, and the elucidation of enzyme specificity and protein-ligand interactions [18,19]. The success of macromolecular X-ray crystallography in understanding the molecular mechanisms underlying diseases and metabolic disorders is well documented. For example, X-ray crystallography has played a pivotal role in elucidating the structures of hemoglobin and its allosteric changes, which is directly relevant to understanding sickle cell disease and to better comprehending cardiovascular health [20]. Beyond hemoglobin, X-ray crystallography has also provided valuable insights into the structure and function of numerous other disease-related proteins, such as enzymes, receptors, and signaling molecules. These structures have enabled structure-based drug design efforts targeting a wide range of diseases and metabolic disorders [21,22]. The continued advancements in X-ray crystallography techniques, including improvements in sample preparation, data collection, and computational methods, have expanded the scope and applicability of this technique in structural biology and drug discovery [23].

The way in which enzymes perform their function implies constant changes in shape. To observe and record this well-choreographed process on the molecular scale is one of the most important challenges currently facing structural biology, and traditional X-ray crystallography cannot overcome it [24]. Conventional macromolecular X-ray crystallography is performed on single protein crystals kept at cryogenic temperatures [25]. The freezing conditions perturb the enzymes ‘native state’ and confer a static character on the technique [26]. In a nutshell, the protein is regarded through a snapshot taken in a specific time point. Moreover, it is necessary to rotate the crystal to completely expose it to the X-ray beam and collect data from the whole crystal. These long exposure times increase the radiation-induced damage and can produce artifacts, particularly specific damage to the side chains of molecules within the crystal [27]. As will be discussed in the following sections, serial crystallography (SX) was developed to overcome the limitations of traditional X-ray crystallography. This technique collects data from micrometer-sized crystals, minimizing the radiation damage by capturing diffraction images from individual crystals. This approach follows the principle known as ‘diffraction before destruction’ [28]. Furthermore, time-resolved serial crystallography (TR-SX) extends SX to study macromolecular dynamics using the pump-probe principle, allowing for investigation of protein structural changes over various timescales [29].

## 4. Microcrystals Are Ideal for Studying Protein Dynamics and Catalysis of Metabolic Diseases

Throughout the history of macromolecular X-ray crystallography, many scientists have asked, are crystals physiologically relevant, that is, ‘*native-like*’? Enzyme, and overall protein, crystals tend to be seen as static entities and therefore unsuitable for studying enzyme catalysis, given that this involves conformational changes. However, it has been known for a long time that enzymes remain catalytically active within crystals [30], although the environment within a crystal lattice has little resemblance to the actual cellular neighborhood of a protein. Proteins are highly dynamic molecules, but even though the lattice restrains their accessible conformational space, they retain a remarkable degree of plasticity [31] and often display surprisingly large-scale conformational changes during ligand binding. The accessible conformational space depends on how the protein is packed in a given crystal form, and analysis of multiple crystal forms provides a more complete picture of the conformational landscape [32]. Recently, new methods have been devised that explicitly take advantage of the structural plasticity of proteins in crystals to study allostery. Ranganathan and *co-workers* monitored the structural changes caused by an electric field through time-dependent Laue diffraction experiments [33], whereas Fraser and colleagues have established multi-temperature crystallography to reliably map allosteric networks by defining and explicitly modeling structural elements that populate different conformations as the data collection temperature is shifted in steps from the standard 100 K to 273–310 K [34].

Macromolecular crystals are typically about 50% protein and 50% solvent, which is like the 65% fraction of water within human cells. Analyzing more than 36,000 enzymes listed in the BRENDA database reveals that (a) the median turnover time for catalysis in solution is about 70 ms, (b) more than 60% exhibit a k_cat_ value between 1 and 100 s^−1^, and (c) enzymes catalyzing reactions related to secondary metabolism are typically 30-fold slower than those of the central metabolism [35,36,37,38]. Dynamics play important but often ill-defined roles in enzyme catalysis [39,40]. A driving hypothesis for time-resolved serial crystallography is that because small molecule substrates diffuse relatively quickly, enzyme microcrystals will equilibrate with substrates faster than catalytic turnover [41,42]. Reactions in crystals must be synchronized throughout all the unit cells to observe high-resolution diffraction from reaction cycle intermediates. Due to their high surface area-to-volume ratio, microcrystals can indeed be very advantageous for studying enzyme dynamics and catalysis of enzymatic reactions, as it enhances the enzyme-substrate interactions and catalytic event observations. Their shorter diffusion time for substrates and products facilitates faster substrate turnover, aiding in enzyme kinetics studies. Improved access to enzyme active sites in microcrystals is particularly beneficial for enzymes with buried active sites, allowing for a more accurate representation of the catalytic activity. Additionally, their smaller size enables quicker reaction times, making it possible to capture transient intermediates and observe the enzyme’s catalytic cycle *in real-time*.

## 5. Time-Resolved Serial Crystallography

X-ray free-electron laser (XFEL) facilities were introduced as a revolutionary scientific-technical advancement in 2009 [43]. With this extraordinarily powerful technology, a new paradigm in structural biology became available. XFEL has revolutionized crystallography by allowing data collection from nano- and microcrystals [28] and room temperature data collection without the limitations of radiation damage [28,44]. The advent of XFELs removes many obstacles in the path toward serial noncryogenic crystallography. This progress was made possible by the availability of new XFEL sources coming into play, as well as by associated advances in sample delivery [45] and data processing [34,46,47]. XFELs allow the circumvention of cryocooling to ameliorate radiation damage, since the phenomenon of ‘diffraction before destruction’ enables damage-free room temperature data collection [28,44]. For this reason, XFELs could straightforwardly be used for experiments at multiple temperatures in the regime above the glass transition (>180–220 K); for example, for nanocrystals to microcrystals that are too small or otherwise not amenable to synchrotrons.

A great advantage of XFELs is that they enable access to the time dimension. The ultrafast (femtosecond) timescale and extreme brightness of XFEL pulses also allow the collection of series of datasets related by time delays, offering insights into phenomena such as enzyme dynamics and catalysis. Time-resolved series of datasets (see reviews [48,49]) can directly visualize protein motions that may be relevant to function. This method is an attractive alternative to time-resolved Laue crystallography, which has stringent technical limitations on parameters such as the crystal mosaicity. Many time-resolved XFEL studies use photoactivatable proteins, but recent and near-future developments will vastly broaden the scope of these experiments (see reviews [48,49]). Additionally, many XFEL-related developments are trickling down to synchrotrons [50], which will broaden their applicability in the coming years, when synchrotrons will still outnumber XFELs. However, new methods are being added to the time-resolved XFEL toolkit that will move the field beyond this limitation, allowing much wider-reaching explorations of how protein structures dynamically respond to perturbations. For example, as presented in the next section, the ‘mix-and-inject’ strategy takes advantage of the small crystals that can be analyzed with XFELs to rapidly soak in ligands and initiate biochemical reactions in the crystals [49,51,52,53,54,55].

## 6. Metabolic Reactions with Time-Resolved Serial Crystallography

Enzyme dynamics in metabolic disease is a prominent area of biophysics research, exploring how enzymes transition between different conformations during their function. Enzymes, like other proteins, exist within conformational ensembles, and these dynamics are crucial for their catalytic activity. Observing the catalytic reaction of an enzyme in atomic detail coupled to allosteric and cooperative effects has been a long-standing dream of structural biologists since the first structure of an enzyme was solved [56]. Time-resolved X-ray crystallography (TRX), pioneered by Laue crystallography, has advanced our ability to observe proteins’ non-equilibrium behavior during catalysis [49,53,57,58]. The recent demonstrations of the time-resolved imaging of enzyme catalysis under near-physiological conditions [51,52,53], made possible by XFEL crystallography, provided impressive insights into the new field of structural enzymology. Since almost all aspects of life are controlled by enzymes, knowledge of the dynamics of these reactions will have a high impact on designing inhibitors of common diseases threatening our health. Using serial crystallography with XFELs or synchrotron sources, researchers can track protein responses to perturbations, such as substrate binding or light activation, with high precision and reduced radiation damage. This approach has revealed that catalysis can induce enzyme motions that are not evident without substrates. However, interpreting the data, especially identifying minor conformations crucial for catalysis and protein entropy, remains challenging. Strategies to enrich and study these minor conformations are essential for understanding the reaction coordinate fully. TR serial femtosecond X-ray crystallography (TR-SFX) studies have been demonstrated with photoinduced reactions or with proteins and their respective ligands or substrates.

Conformational dynamics drive ubiquitous protein functions, such as enzyme catalysis, signal transduction and allosteric regulation [59]. The study of protein motions is critical to our mechanistic understanding of these fundamental biological phenomena, and the ability to rationally control protein motions is a frontier for protein engineers who seek to design biomolecules with increasingly complex functions. Nevertheless, accurately modeling protein motions with atomic resolution remains a longstanding challenge for the field of structural biology [60]. This challenge arises because data from imaging techniques such as X-ray crystallography are inherently ensemble-averaged over space and time [61]. Even though macromolecular crystallography is by far the most successful technique used by the research community (more than 186,000 atomic models were deposited in the PDB as of July 2024), the vast majority of the macromolecular crystallography datasets are collected from frozen samples. Thus, information about the spatiotemporal coupling between the observed alternate conformations is lost, and intermediate states that are only transiently populated at equilibrium remain invisible. For this reason, high-resolution structural information is often combined with spectroscopic measurements. Despite the power of such integrative approaches, it can be difficult to correlate spectroscopic observables with specific features of molecular structures. TRX overcomes all these challenges by obtaining high-resolution information in both the spatial and temporal domains [33,62]. In this pump-probe technique, a rapid perturbation drives molecules out of conformational equilibrium, then structural snapshots are captured as the ensemble relaxes to a new equilibrium. By sampling a series of pump-probe time delays, the kinetic couplings between conformers and transiently populated structural states can be observed.

**Figure 2:**
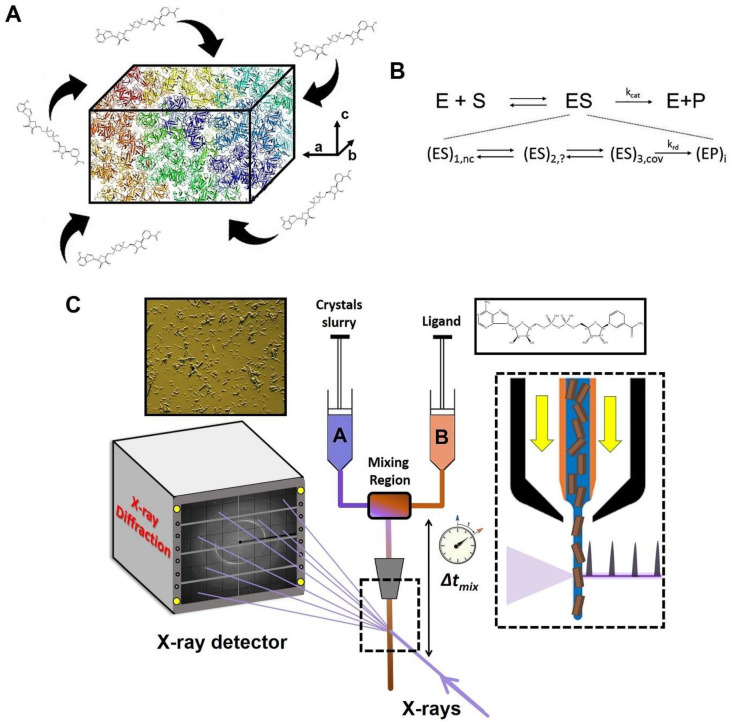
Concept and experimental setup of a mix-and-inject serial crystallography (MISC) experiment. (**A**) Enzyme microcrystals are mixed with substrate (here, an NADH molecule). The substrate diffuses into the crystals and starts the enzymatic reaction to be probed by X-ray pulses at various mixing delays. (**B**) Upper row, steady state, no time resolution: simplified catalytic mechanism introduced by Michaelis-Menten. Second row, transient-state kinetics with time resolution: substrate binds non-covalently (nc) to the enzyme. The reaction proceeds through intermediates, which may (or may not) consist of a covalently (cov) bound substrate. The substrate is catalytically modified to a product with several enzyme-product intermediates. A rate-determining step parametrized by a rate coefficient k_rd_ determines k_cat_. Arrows depict chemical reactions characterized by rate coefficients. (**C**) In an MISC experiment, a slurry of enzyme microcrystals is rapidly mixed with a substrate solution in a continuous flow-mixing device, initiating the reaction. As the reaction progresses, the crystals flow through the X-ray interaction region in a freestanding microfluidic jet, where they are probed by a pulsed X-ray source. By varying the distance between the mixer and the interaction region, different time points of the reaction can be measured.

TRX experiments were pioneered on photoactive proteins [63,64,65] and have become more readily accessible with the advent of XFELs, brighter synchrotrons and serial crystallography [66,67,68]. The scope of TRX has been broadened using photocaged ligands [69], electric-field-based perturbations [33], and rapid mix-and-inject experiments, with the latter being one of the most important developments in relation to investigating the structures and functions of enzymes with both XFELs [49,52,53,54,70,71] and synchrotrons [72]. The mix-and-inject technique, commonly known as MISC, employs diffusion to initiate a reaction [42]. Serial crystallography at intense pulsed X-ray sources in combination with suitable mixing injectors [73] enables this technique. Reactions can be started simply by diffusion of substrates or ligands into the microcrystals, as shown schematically in Figure 2A. Since diffusion times depend on the crystal size, very small crystals must be employed to swiftly initiate enzymatic reactions much faster than the turnover time. As a result, reactions in enzymes and other interesting biomolecules can finally be observed *in real time*. With MISC, the initial (pre-steady state) phase of substrate binding and processing is explored. Typically, after the formation of a non-covalently bound enzyme-substrate complex, the structures of one or multiple intermediate states may be determined. As can be seen from Figure 2B, the steady state Michaelis complex (ES) consists of a mixture of several intermediates weighted by their respective occupancies. Non-covalently and covalently bound enzyme-substrate complexes, as well as covalently and non-covalently bound enzyme-product complexes with catalytically modified substrate molecules, can all be present at the same time. MISC can be used to unravel the structures of all or a subset of these complexes depending on the kinetic mechanism. Figure 2C illustrates the experimental setup of a typical MISC experiment.

**Figure 3:**
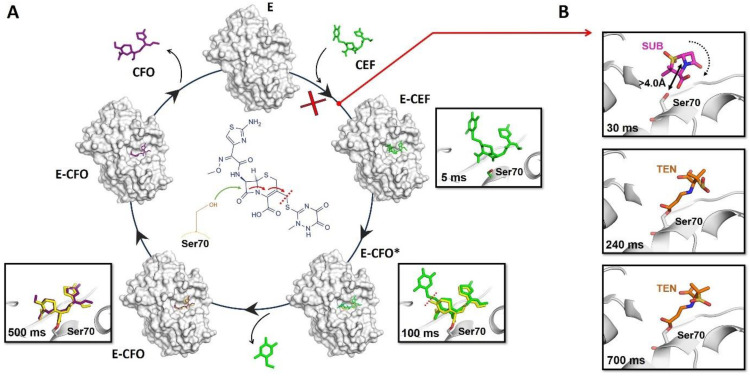
Schematic of the catalytic and inhibition mechanisms of the BlaC enzyme. (**A**) Inactivation mechanism of the antibiotic ceftriaxone (CEF) by the BlaC enzyme. All the states shown in the mechanism were captured by TR-SFX. The formation of the complex E-CEF occurs just 30 ms upon reaction initiation. As shown in the middle panel, the nucleophile attack, opening of the beta-lactam ring with the subsequent covalent bond formation between the Ser70 and a shortened specie (E-CFO*), and the release of the leaving group occur at time points between 100 and 500 ms. (**B**) Inhibition mechanism of BlaC by the inhibitor sulbactam (SUB). The formation of the complex E-I occurs 30 ms upon mixing. The nucleophilic attack by Ser70 opens the lactam ring of SUB, leading to the formation of acyl-enzyme intermediate (TEN) in a *trans* configuration 240 ms later.

In the past seven years, several groundbreaking experiments have been performed and they have demonstrated that the MISC method can be used to observe transient intermediates of enzymes engaged in catalysis [49,51,52,53,54,70,71,74]. As an example of this success, a recent study carried out with BlaC, a beta-lactamase enzyme from *Mycobacterium tuberculosis*, reports how the enzyme catalyzes the ring cleavage of ceftriaxone, a so-called third-generation cephalosporin antibiotic [53,54]. The authors collected a number of datasets at time points ranging from 3 to 500 ms after mixing with the antibiotic [53,54]. The resulting electron density maps revealed features that were modeled as reaction intermediates that build up and go away with the reaction time after mixing. From these experiments, the authors were able to capture the exact moment at which the enzyme attacks the antibiotic, leading to the opening of the beta-lactam ring, thus inactivating it (Figure 3A). As a complement to this study with BlaC, in a more recent experiment, it has been visualized, *in real time*, how the shape of the catalytic site of this enzyme determines its inhibition mechanism by sulbactam [49]. This study revealed ligand-binding heterogeneity, ligand gating, cooperativity, induced fit, and conformational selection, detailing how the inhibitor approaches the catalytic clefts and binds to the enzyme noncovalently before reacting to a *trans*-enamine [49] (Figure 3B). The combined results of these two experiments align with the practice of administering the antibiotic ceftriaxone alongside the inhibitor sulbactam. This successful approach allows sulbactam to inhibit BlaC before it can deactivate ceftriaxone. Consequently, ceftriaxone can effectively target and inactivate penicillin-binding proteins.

## 7. Examples of How Time-Resolved Serial Crystallography Studies Can Help Fight Metabolic Diseases

Time-resolved serial crystallography (TR-SX) offers valuable insights into the dynamic processes of enzymes involved in metabolic diseases. By capturing snapshots of enzyme-substrate interactions over time, researchers can uncover the molecular mechanisms underlying enzyme function and dysfunction, identify transient conformations critical for catalysis, and visualize the structural changes associated with enzyme activation [52,53,54]. This information is crucial for guiding drug design, optimizing inhibitors, and enhancing the efficacy of therapeutic interventions. Furthermore, TR-SX provides detailed structural information on enzyme-ligand interactions, aiding in the development of more potent and selective drugs for metabolic disorders. Below, we discuss examples of key proteins essential for human metabolism, highlighting how TR-SX can improve our understanding of their functions and facilitate the creation of effective inhibitors. We have divided the examples below into two subsections: those proteins that have already been studied extensively using TR-SX and those that show significant potential for future TR-SX experiments.

### 7.1. Human NAD(P)H:Quinone Oxidoreductase 1 (NQO1)

Human NAD(P)H oxidoreductase 1 (NQO1) is a crucial flavoenzyme involved in the reduction of quinones to hydroquinones, a process that prevents the formation of reactive oxygen species (ROS) and shields cells from oxidative stress [75]. NQO1 plays a significant protective role in cardiovascular health by maintaining redox homeostasis and mitigating oxidative damage, which are essential to preventing cardiovascular diseases such as atherosclerosis, hypertension, and myocardial infarction [76]. The enzyme’s function extends to modulating inflammatory responses, which are pivotal in the progression of cardiovascular disorders. Additionally, NQO1 stabilizes tumor suppressor proteins and influences various signaling pathways vital for cellular defense mechanisms [77]. Genetic polymorphisms and reduced expression of NQO1 have been linked to an increased risk of cardiovascular diseases, underscoring its importance in cardiovascular health [78]. Enhancing the activity of NQO1 or mimicking its protective effects presents a promising avenue for developing novel therapeutic strategies aimed at treating cardiovascular conditions [79]. By leveraging the enzyme’s natural protective functions, such therapies could significantly reduce the burden of cardiovascular diseases, offering new hope for patients [78].

Its multi-activity and effects on several biochemical pathways make NQO1 an attractive target for drug discovery, and a deeper knowledge of its structure and mechanism can reveal the dynamics during the catalytic function of NQO1. Structurally, NQO1 is a homodimer with two identical 31 kDa interlocking subunits and two active sites located at the interface [80,81,82,83]. The two active sites contain residues from both subunits and each one is occupied by the FAD cofactor and is bound by the NAD(P)H substrate in such a way that the nicotinamide ring lies parallel to the FAD, facilitating efficient electron transfer [81]. NQO1 catalyzes the reduction reaction of quinones to hydroquinones by a double-displacement mechanism commonly known as a ‘ping-pong’ mechanism. The full reaction consists of (a) binding and oxidation of the electron donor NADH by FAD that is reduced to FADH_2_ with NAD^+^ leaving the binding site; and (b) binding and reduction of the substrate by the FADH_2_ [84,85,86].

**Figure 4:**
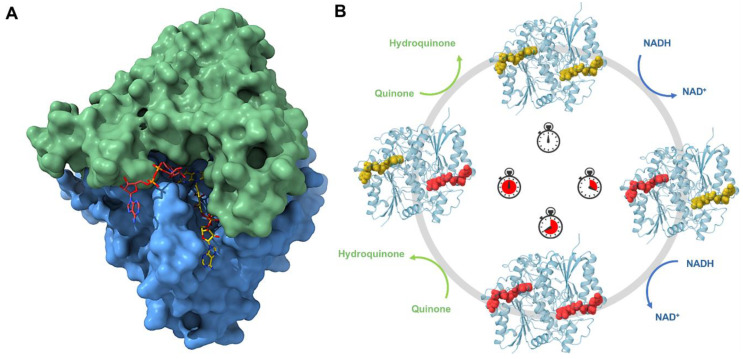
Structure and mechanism of NQO1. (**A**) Surface representation of NQO1’s homodimer bound to NADH. The panel below shows a closer view of the catalytic site. (**B**) Schematic of the plausible mechanism for the reductive and oxidative half-reactions. Due to the cooperativity described for human NQO1, the reduction and the oxidation of the enzyme occur in a sequential manner and the oxidative half-reaction does not take place until the two catalytic sites of the enzyme are fully reduced.

So far, structure biology has helped to obtain all this information and previous crystal structures with and without inhibitors have shown that NQO1 undergoes local conformational changes in which each active site opens and closes during the reaction [80,81,87,88]. However, conventional macromolecular crystallography with synchrotrons has limitations in capturing structural and dynamic information on many intermediate states, which hinders a comprehensive understanding of the redox mechanisms. This approach is not suitable for studying fast conformational changes and rapid reactions, such as those involved in redox processes. To overcome this hurdle, novel approaches can be used, such as room temperature serial crystallography with synchrotrons and with XFELs. In this regard, with the attempt to decipher the complex catalytic mechanism of NQO1, the first structure at room temperature of NQO1 at the Linear Coherent Light Source (LCLS) has been solved [89]. These results revealed for the first time the key roles of Tyr128 and Phe232, which have an unexpected flexibility within the crystal at room temperature. For the first time, it was possible to observe the conformational heterogeneity of these residues and the high plasticity of NQO1. In order to develop new insight into the flavin reductive half-reaction of the catalytic mechanism of NQO1, serial synchrotron crystallography (SSX) has been recently carried out by our team to determine the structure of the NQO1 in complex with NADH [55] (Figure 4A). Before this, no structure of NQO1 in complex with NADH had ever been deposited in the PDB. Our structural and MD simulation results provided the first structural evidence that NQO1’s functional negative cooperativity is driven by structural communication between the active sites through long-range propagation of cooperative effects across the protein structure, as well as supporting the notion that the binding of NADH significantly decreases protein dynamics and stabilizes NQO1, especially at the dimer core and interface [55].

Both these studies [55,89] were crucial to showing the benefits of room temperature experiments. The use of serial crystallography with microcrystals has allowed the possibility of analyzing this protein under some aspects that were unknown before. Going on with the study of the protein dynamics occurring in the reductive half-reaction at different time points could reveal different intermediates along the reaction coordinate. The implementation of a femtosecond XFEL timescale could help even more to study the mechanism at shorter time points. The final goal is definitely showing its role as an antioxidant and as a potential target to treat common diseases in which NQO1 is involved, advancing the design of new, more potent, and effective inhibitors that can be used in a clinical setting (Figure 4B).

### 7.2. Glutaminase C (GAC)

One hundred years ago, Otto Warburg discovered for the first time an altered metabolic pattern in cancer cells in contrast to normal ones. Since then, this phenomenon has been known as the ‘Warburg effect’, and it consists of an uncoupling of the glycolytic pathway from the tricarboxylic acid (TCA) cycle. As a result, cancer cells rely on an elevated glutamine metabolism to meet their energetic and biosynthetic requirements. More than half of the TCA metabolites are generated by glutamine as a precursor in the metabolic pathway known as glutaminolysis [90]. This ‘glutamine addiction’ of cancer cells is supported by an overexpression of the mitochondrial enzyme glutaminase, which catalyzes the first step of glutaminolysis. Glutaminase converts glutamine into glutamate, which is subsequently converted into the TCA cycle intermediate α-ketoglutarate [91]. There exist two isozymes of mammalian glutaminase: kidney-type glutaminase and liver-type glutaminase, each of which has two splice variants, a shorter and a longer isoform. The C-terminal truncated splice variant of the kidney-type glutaminase is named glutaminase C (GAC), and it has been directly implicated in the progression and survival of numerous aggressive cancers [92]. Thus, targeting cancer cell-enhanced glutaminolysis via GAC inhibition is emerging as a promising strategy to disrupt tumor progression. Research has focused on the use of small molecules as inhibitors [93].

**Figure 5:**
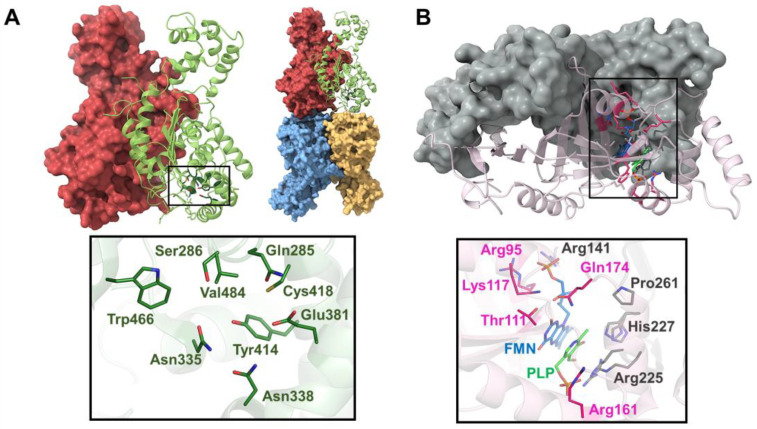
Crystal structures of the GAC and PNPOx enzymes. (**A**) Surface and cartoon representation of the homodimer of the GAC enzyme (upper panel, left) and the GAC tetramer (upper panel, right). Residues at the catalytic site are shown as sticks. A closer view of the catalytic site (black boxed in the upper panel) is shown in the lower panel. (**B**) Surface and cartoon representation of the homodimer of the PNPOx enzyme (upper panel). Residues and the PLP at the catalytic site are shown as sticks. A closer view of the catalytic site (black boxed in the upper panel) is shown in the lower panel.

GAC is a 65 kDa enzyme composed of 598 residues. The first 16 residues of the N-terminal form a mitochondrial localization signal. Following the translocation to the mitochondrion, a segment comprising the first 72 residues of the enzyme is truncated in a post-translational modification. The remainder of the protein consists of three domains, with the central one being catalytic (Figure 5A). GAC may exist as either a dimer or tetramer. Whereas the dimer is inactive, the tetramer has catalytic activity (Figure 5A). The tetramerization of GAC has been seen in vitro upon the addition of phosphate or other polyvalent anions, but it is still unclear how GAC becomes activated in living cells [92].

Many attempts to develop GAC inhibitors have been made during the last few years. Small molecules demonstrated potential as GAC inhibitors, especially in the case of bis-2-(5-phenylacetamido-1,2,4-thiadiazol-2-yl)ethyl (BPTES), which was reported to inhibit GAC via an allosteric mechanism by binding to and stabilizing an active tetrameric state of the enzyme, rather than by competition with glutamine. This finding started a new class of GAC allosteric inhibitors and inspired the design of different analogs with higher potencies, such as CB-839 and UPGL00004, which bind to GAC with much lower IC_50_ values than BPTES [92]. Currently, CB-839 is the lead compound in clinical trials, both in monotherapy and as part of drug mixtures. However, none of the BPTES class of allosteric GAC inhibitors is yet approved for cancer treatment and their mechanisms of action remain unknown [91]. Traditional X-ray crystallography has helped in elucidating the binding site of BPTES, CB-839, and UPGL00004. These compounds bind at the interface of the GAC tetramer by trapping the enzyme in an inactive conformation and regulating its enzymatic activity via a similar allosteric mechanism [91,92]. Nevertheless, despite extensive efforts, traditional X-ray crystallography has been unable to explain the molecular determinants that dictate the basis for the different potencies exhibited by these compounds. However, in a recent study, it has been shown that crystallography experiments at ambient temperature using the serial crystallography technique may be a way to advance inhibitor design for GAC. In this study, Milano and co-workers have observed clear differences between the binding conformations of inhibitors with significantly different potencies [93].

The use of TR-SX could allow researchers to capture structural changes in GAC at various stages of its catalytic cycle, providing high-resolution snapshots of enzyme dynamics in real time. By utilizing TR-SX, scientists can observe how GAC interacts with different inhibitors over time, identifying key binding contacts and conformational changes that contribute to inhibitor potency. This dynamic view will be crucial for understanding the precise molecular determinants that enhance or diminish inhibitor efficacy, which are often elusive in static crystal structures obtained under cryo-cooled conditions. Furthermore, TR-SX can help elucidate the allosteric regulation of GAC by revealing how inhibitors induce conformational changes that modulate enzyme activity. This information will be vital for designing allosteric inhibitors that can effectively disrupt GAC’s catalytic function, paving the way for new therapeutic strategies.

### 7.3. G Protein-Coupled Receptors (GPCRs)

G protein-coupled receptors (GPCRs) are essential components of cell signaling and represent the largest superfamily of cell surface receptors, encoded by approximately 1000 genes. These receptors respond to a diverse range of extracellular stimuli, including photons, ions, lipids, neurotransmitters, hormones, peptides, and odorants. GPCRs consist of a single polypeptide that folds into a globular shape embedded in the cell’s plasma membrane, featuring seven alpha helices that span the membrane. Extracellular loops form binding pockets for signaling molecules [94]. When a signaling molecule binds to a GPCR, it activates G proteins on the intracellular side, triggering the production of second messengers and initiating downstream signaling cascades. Through these mechanisms, GPCRs regulate various physiological functions, such as vision, sensation, growth, and hormone responses. Mutations or alterations in GPCR functionality can lead to dysregulation, contributing to several chronic human pathologies, including cancer, cardiovascular diseases, metabolic disorders (e.g., obesity and diabetes), and immune disorders [95,96,97]. Consequently, GPCRs are highly attractive therapeutic targets. In fact, it is estimated that one-third to one-half of all marketed drugs act by binding to GPCRs.

Despite the promise of structure-based drug discovery (SBDD) and the enormous applicability of GPCRs as therapeutic targets, they present many challenges for SBDD, such as their inherent low expression and high conformational dynamics. The last decade has seen a revolution in the structural biology of clinically important GPCRs. Indeed, recent advancements in protein engineering, X-ray crystallography methods (see below), cryo-electron microscopy (cryo-EM), NMR, and molecular dynamic simulations have revolutionized our understanding of GPCR structures and dynamics. These studies have provided insights into ligand-receptor interactions, conformational changes, and signaling complexes, offering unprecedented opportunities for investigating receptor activation, orthosteric/allosteric modulation, biased signaling, and dimerization [94,98]. Among all the structural biology techniques, cryo-EM and X-ray crystallography have played a significant role in advancing our understanding of GPCRs’ structure and function. Cryo-EM emerged as a powerful tool for GPCR structural determination, particularly for complexes with G proteins and arrestins. It does not rely on crystal formation and can accommodate the flexibility of these complexes. Rapid advancements in cryo-EM technology have made it a viable alternative for determining the structures of even small GPCRs. Regarding X-ray crystallography, despite all the advances in technology, including receptor stabilization and crystallization in a membrane mimetic environment (*in meso* or LCP crystallization), obtaining large, well-diffracting crystals for data collection at synchrotron radiation sources is often a very frustrating process that sometimes may even take, on average, up to several years. In addition, the amount of crystallographic information that one can obtain from well-ordered but small microcrystals at microfocus beamlines at synchrotron sources is very limited by radiation damage. However, these limitations have been essentially avoided using SFX technology with XFELs. In fact, SFX has rapidly accelerated structural studies of GPCRs in the past decade. Since the determination of the first GPCR structure by SFX in 2013, the serotonin 5-HT2B receptor bound to the migraine drug ergotamine [99], this technique has elucidated numerous GPCR structures in various conformational states, bound to diverse ligands, and in complex with their intracellular partners: G proteins and arrestins, thus providing invaluable insights into the molecular mechanisms of receptor activation and ligand binding. The use of receptor engineering techniques, such as fusion proteins, antibody fragments, and thermostabilizing mutations, has been crucial to achieving this goal. SFX has also achieved significant milestones in the field of GPCRs, such as the determination of novel GPCR structures, including the angiotensin II receptors, the melatonin I and II receptors and the prostaglandin E2 receptor [100,101,102,103]; the elucidation of the structure of receptors in complex with their natural binding targets (rhodopsin-arrestin) or monoclonal antibodies (5-HT2B-Fab) [104,105,106] and the structures of full-length GPCRs such as the frizzled smoothened receptor and the class B glucagon receptor [107,108]. For more information about SFX and its applicability to GPCRs and the SBDD field, see two recent comprehensive reviews by Cherezov and co-workers [109,110,111].

The identification of new drugs targeting GPCRs hinges on a detailed understanding of GPCR biology, especially their structural biology, due to the intricate structure-function relationships in GPCR signaling. However, integrative structural biology approaches, combining SFX, cryo-EM and solution NMR, will be essential for fully understanding GPCR mechanisms. These techniques can analyze function-related conformational changes, allosteric coupling, variable efficacy, and biased signaling, all of which are critical for drug development. Advances in structural biology not only support the discovery of new drugs but also enhance the design and application of existing ones. The future goal is to design drugs with specific signal selectivity and molecular properties suitable for clinical use.

### 7.4. Metalloenzymes

#### 7.4.1. Metalloenzymes and Crystallography

Metalloenzymes are key to many metabolic processes in all living organisms, including respiration, photosynthesis, and nitrogen fixation. Metalloenzymes are enzymes that contain one or more metal ions as cofactors, which provide proteins with an enriched functional range by expanding their physiochemical properties and playing a role in activation and stabilization during catalysis [112,113]. It has been estimated that about one-third of proteins require metal ions to perform their function [114], and about 30–40% of enzymes contain transition metals or metallocofactors at their catalytic centers, such as heme, iron−sulfur (Fe−S) clusters, and cobalamin (Cbl) [115]. These metals act as cofactors involved in enzymatic reactions and catalytic functions, including ubiquitous proteins such as superoxide dismutase, carbonic anhydrase and alcohol dehydrogenase. Despite the success of traditional X-ray crystallography in revealing the reaction mechanisms and thus the function of metalloproteins, this technique suffers from several important challenges, including protein preparation since the correct metal ion must be incorporated into the protein. Moreover, it can be challenging to obtain large, well-ordered crystals. Finally, these protein crystals tend to be susceptible to radiation damage, which is caused by X-ray-generated free radicals. The latter is probably the most critical limitation in structural studies of metalloproteins by X-ray crystallography. Photoreduction, radiolysis and ionization deriving from the X-ray beam used to probe the structure complicate structural and mechanistic interpretation. The interaction with X-rays modifies the sample under study over the course of X-ray exposure, and therefore, the information is generally always that of an X-ray-modified structure or a mixture of the native and modified states [116,117,118]. This radiation damage occurs by absorption of X-ray photons by the atoms in the sample. The spread of radiation damage can occur due to different mechanisms, with the most prominent being the generation of free radicals that can then migrate and create a cascade of redox/damage processes, and it was found that the amount of radiation damage observed is predominantly dependent on the dose and not on the irradiation time [119].

This situation changed with the advent of XFELs and, particularly, through the TR-SFX technique, which offers a tool to probe such labile systems with high X-ray doses in an ultra-fast fashion, extracting the information from very small crystals (nano- and micro-crystals) before manifestation of X-ray-induced changes to the sample, i.e., outrunning the slower damage processes by ultrafast data collection [28,119]. Overall, studying metalloenzymes with TR-SFX offers a powerful approach to unraveling their dynamic behavior, catalytic mechanisms, and regulatory processes at the molecular level. This knowledge not only advances our understanding of enzyme function and regulation but also holds promise for drug discovery and the development of new therapeutic strategies for treating diseases. Owing to the rapid reactions and sensitivity to radiation damage of metalloenzymes, they cannot be studied effectively by traditional X-ray crystallography. One of the major advantages of TR-SFX experiments in the field of metalloenzymes is that they can be coupled with X-ray emission spectroscopy (XES) experiments. By studying metalloenzymes with TR-SFX/XES at XFELs, researchers can gain insights into the precise arrangement of metal ions, ligands, and active site residues, providing a detailed understanding of the enzyme’s catalytic mechanism. This information is invaluable for drug design, biotechnology applications, and understanding fundamental biochemical processes. In this regard, to date, there have been several notable examples of metalloenzymes investigated using serial SFX at XFELs, including the following. (1) Photosystem II (PSII): PSII is a metalloenzyme involved in the oxygen-evolving photosynthesis pathway. XFELs have been used to study PSII crystals to capture the transient state of water oxidation, which is facilitated by a cluster of four manganese ions [120,121,122]. (2) Cytochrome c Oxidase: cytochrome c oxidase is a metalloenzyme found in mitochondria that plays a crucial role in the electron transport chain. Studies using XFELs have provided insights into the reaction intermediates and oxygen reduction mechanism of this enzyme, which contains a heme iron and copper centers [71,123]. (3) Nitrogenase: nitrogenase is a metalloenzyme responsible for the biological conversion of atmospheric nitrogen into ammonia. XFEL studies have been used to investigate the active site of nitrogenase, which contains a complex iron-molybdenum cofactor (FeMo-co) or iron-vanadium cofactor (FeV-co), depending on the organism [124]. (4) Carbonic Anhydrase: carbonic anhydrase is a metalloenzyme that catalyzes the reversible hydration of carbon dioxide. XFEL studies have provided insights into the proton transfer mechanism and dynamics of the Zn^2+^ ion bound active site in carbonic anhydrase [125]. These are just a few examples, and research in this field is ongoing, with new metalloenzymes being investigated using XFELs to elucidate their structures and catalytic mechanisms.

#### 7.4.2. Metalloenzymes in Metabolic Diseases

However, to the best of our knowledge, there may not be specific examples of metalloenzymes involved in metabolic diseases that have been investigated using SFX with XFELs so far. In addition to their catalytic efficiency, metalloenzymes play critical roles in various metabolic pathways, and the dysregulation of these enzymes can contribute to the development of metabolic diseases. There are many examples of metalloenzymes and their involvement in metabolic diseases, such as the following. (1) Phenylalanine hydroxylase (PAH), which is an iron-containing metalloenzyme that catalyzes the conversion of L-phenylalanine to L-tyrosine, a reaction essential for the metabolism of phenylalanine [126]. Mutations in the PAH gene cause phenylketonuria (PKU), a metabolic disorder characterized by elevated phenylalanine levels [126]. (2) Dipeptidyl peptidase-4 (DPP-4), which is a zinc-containing metalloenzyme involved in the degradation of incretin hormones such as glucagon-like peptide-1 (GLP-1) and glucose-dependent insulinotropic polypeptide (GIP) [127]. Inhibition of DPP-4 is a therapeutic target for type 2 diabetes mellitus [127].

Specific examples of TR-SFX studies directly investigating metabolic diseases are limited. After a thorough search of the literature, currently and to the best of our knowledge, there is no study on metalloenzymes associated with metabolic diseases that has been carried out using TR-SFX. There are several good reasons to look at metalloenzymes with TR-SFX:

1. Capturing Transient States: Metalloenzymes often undergo conformational changes and structural rearrangements during catalytic cycles or upon substrate binding. Capturing the transient states and intermediate structures of metalloenzymes with high temporal resolution will provide us with insights into reaction mechanisms and dynamics.
2. Visualizing Metal Ion Coordination: Metal ions are crucial to the catalytic mechanisms of metalloenzymes, influencing coordination geometry, ligand-binding dynamics, and redox states. Understanding these factors is essential for gaining insights into the enzymes’ catalytic activities and regulatory mechanisms.
3. Probing Metallocofactor Dynamics: Many metalloenzymes feature metal cofactors such as iron-sulfur clusters, heme groups, or metal ions that undergo redox reactions or structural changes during enzyme turnover. Tracking the dynamics of these metallocofactors in real time can reveal how they interact with substrates, cofactors, and inhibitors during catalysis.
4. Uncovering Allosteric Regulation: Metalloenzymes are often regulated by allosteric effectors or environmental factors that influence their activity and specificity. Understanding how allosteric communication pathways and conformational changes are triggered by ligand binding or environmental cues is essential for gaining insights into enzyme regulation and function.
5. Drug Discovery and Design: Metalloenzymes are prime targets for drug discovery due to their critical roles in various biological processes and disease pathways. Revealing atomic-level details of enzyme-substrate interactions, inhibitor-binding modes, and allosteric sites can significantly aid in structure-based drug design, guiding the development of selective and potent enzymatic inhibitors for therapeutic intervention.
6. Understanding Disease Mechanisms: Dysregulations or mutations in metalloenzymes are linked to various diseases, including cancer, neurodegenerative disorders, and metabolic conditions. Understanding the structural and dynamic consequences of these disease-associated mutations can offer mechanistic insights into disease pathology and identify potential targets for therapeutic intervention.

In this review, we will focus on the human cytochrome P450 (CYP450) enzymes [128]. CYP450 enzymes are a superfamily of heme-containing enzymes involved in the metabolism of endogenous (e.g., hormones, fatty acids) and xenobiotic compounds (e.g., drugs, toxins) [128]. Altered activity or expression of specific cytochrome P450 enzymes can influence drug metabolism, hormone levels, and lipid metabolism, potentially contributing to metabolic diseases such as diabetes, obesity, and dyslipidemia [129,130]. Thus, understanding the role of CYP450 metalloenzymes in metabolic diseases can provide insights into disease mechanisms and identify potential therapeutic targets for intervention. Further research into the regulation and function of these enzymes is essential for developing effective treatments for metabolic disorders.

#### 7.4.3. The Human Metalloenzyme Cytochrome P450 2D6 (CYP2D6)

As new drugs reach the marketplace and patients take an increasing number and variety of pharmaceutical agents for a host of medical conditions, the potential for serious drug interactions grows. The rate-determining step at the drug discovery stage is turning a lead molecule into a drug candidate. The strategy used to eliminate or reduce this rate-determining step’s impact is to fully understand the metabolism of drug candidates as early as possible in the drug discovery pipeline. The main drug-metabolizing enzymes in humans belong to the cytochrome P450 (CYP) enzyme superfamily, which is actively involved in the metabolism of most therapeutic drugs and xenobiotics [128]. CYPs have more than 50 polymorphisms, with CYP2D6 being the most abundant isoform and one of the main CYPs contributing to drug metabolism, being responsible for 30% of clinically used drugs currently on the market [131,132]. Furthermore, due to the multitude of pharmacokinetic interactions involving CYP2D6, research on this enzyme is crucial. Therefore, a thorough understanding of the structure and dynamics of CYP2D6 could be an asset in the development of safe and effective drugs.

**Figure 6:**
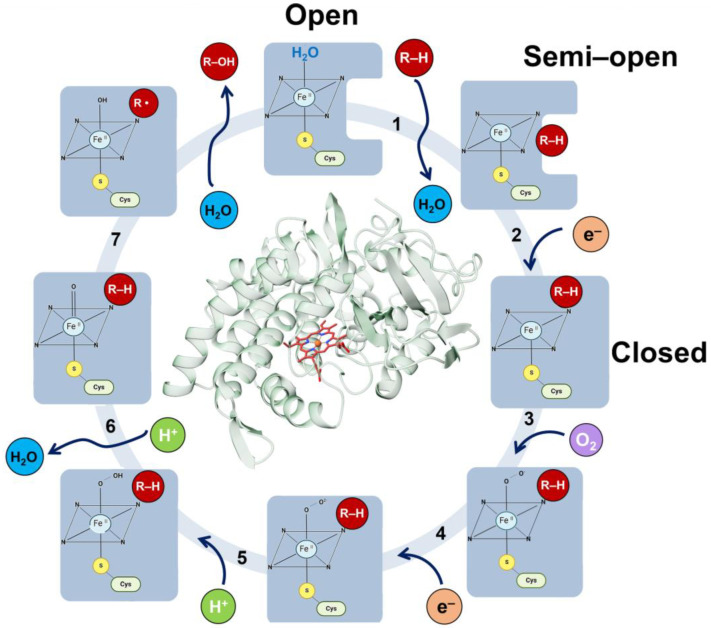
Seven-step mechanism of CYP enzymes. Initially, the heme iron is coordinated to a water molecule and is in a low-spin ferric resting state. (Step 1) The substrate enters the active site, displacing the water molecule and converting the heme iron to a high-spin FeIII state. (Step 2) This change increases the redox potential, allowing electron transfer from the reducing partner to form the ferrous FeII complex. (Step 3) The ferrous FeII complex binds O_2_, forming the oxyferrous complex. (Step 4) The oxyferrous complex is reduced to the peroxo complex. (Step 5) Water molecules return, forming a channel that protonates the peroxo complex to give Compound 0 (Cpd 0). (Step 6) Cpd 0 accepts an extra proton, releasing a water molecule and forming Compound I (Cpd I). (Step 7) Cpd I abstracts a hydrogen from the substrate, leading to its oxidation. The product leaves the pocket, and a water molecule re-coordinates with the heme iron, returning the enzyme to its initial resting state. All the steps are highlighted with black numbers. The enzyme open, semi-open, and closed conformations at each stage are denoted by a blue shape. The substrate, hydrogens, electrons, oxygen, and water are denoted by red, green, orange, violet, and cyan circles, respectively.

The CYP active site contains the heme group (Figure 6), which is situated in the protein’s hydrophobic core and linked to the enzyme surface by channels through which substrates, water molecules, molecular oxygen and products move. The heme group forms the reactive center by activating molecular oxygen and oxidizing the substrate. CYP enzymes function as monooxygenases through an overall canonical reaction mechanism that comprises the reductive scission of the molecular oxygen bond to release one molecule of water with the transfer of a single oxygen atom to the substrate [133,134]. The CYP catalytic cycle has multiple reactions with transient intermediates (Figure 6), where the product of one reaction is the substrate for the next reaction by the same enzyme. Despite the wealth of structural and biochemical information, many details of the complex chemical mechanisms by which these enzymes can activate oxygen have not yet been established. In fact, as of December 2023, over 180 structures of CYPs are deposited in the PDB. Among them, the enzyme CYP2D6 has the second largest number of structures available, with 14 structures either in an open (free enzyme) or closed conformation (bound to substrates or inhibitors). This information deficiency hinders attempts to understand the changes in the drug metabolism due to specific variations. In particular, the mechanism of activation of the bound oxygen molecule and the nature of the activated oxygen species remain uncertain. An understanding of the mechanism of action of these enzymes through CYP2D6 will be invaluable to the advancement of precision medicine. To obtain a better understanding of the structural basis of the enormous catalytic power and biomedical relevance of CYP enzymes, determining the crystal structures of the metastable species on the CYP2D6 reaction pathway will be crucial. To this end, acquiring atomic-level knowledge of the reaction mechanisms and dynamics of the CYP2D6 enzyme within the cytochrome P450 system will help practitioners avoid potentially serious adverse drug interactions.

### 7.5. Human Alanine-Glyoxylate Aminotransferase (AGT)

The human alanine-glyoxylate aminotransferase (AGT) is a peroxisomal pyridoxal 5′-phosphate (PLP)-dependent enzyme that belongs to the class II aminotransferases. AGT is mainly expressed in the liver and catalyzes the transamination between L-alanine and glyoxylate to produce pyruvate and glycine in the presence of PLP, a reaction crucial for glyoxylate detoxification [135,136]. Malfunctions in AGT activity are responsible for a genetic condition known as primary hyperoxaluria type I (PH1), a rare inborn metabolic disease that results in high oxalate production [137]. Oxalate does not seem to be toxic to hepatocytes, but mammals are not able to metabolize it, and it can only be filtered by the glomerulus and secreted by the renal tubes. This leads to a progressive degradation of the kidneys because of the calcium oxalate accumulation until the final loss of renal function [136]. Also, the build-up of oxalate in the body, known as oxalosis, quickly results in severe complications in the bone, heart, skin and kidneys, which are life threatening [138,139]. The most common and efficient treatment or cure for PH1 is double liver and kidney transplantation [140], which is a very invasive and problematic procedure. Therefore, it is clear how PH1 treatment is still challenging and a more specific study of the AGT enzyme functionality and possible defects is needed for advancing the drug discovery.

**Figure 7:**
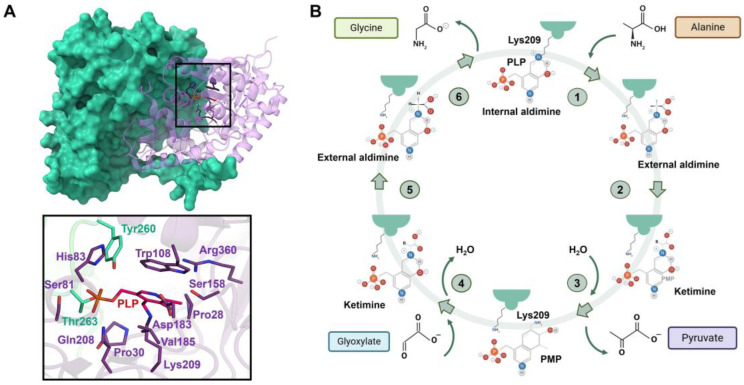
Structure of AGT and scheme of the transamination reaction catalyzed by AGT. (**A**) Upper panel shows the structure of the AGT homodimer, with the residues at the catalytic site shown as sticks. Lower panel shows the interactions that stabilize the Schiff base at the catalytic site. (**B**) In a preliminary step, a conserved Asp residue (Asp183) protonates the pyridine nitrogen N1 of PLP. The first half-reaction (in purple) occurs through steps 1–3: (1) The transaldimination-action with the amino acid substrate only occurs when the internal aldimine is protonated at Nε. (2) The 1,3-prototropic shift represents the rate-limiting step of the first half-reaction. The amino group of the Lys residue acts as a base catalyst extracting the Cα hydrogen. (3) Hydrolysis of the resulting ketimine yields pyridoxamine phosphate (PMP) and the leaving α-keto acid. The second half-reaction occurs through the same steps (4–6) as the first half-reaction in inverse order.

AGT is a homodimeric enzyme in which each monomer is a 43 kDa subunit of 393 residues consisting of a large N-terminal domain (residues 22–282) and a smaller C-terminal domain (283–392) [141,142] (Figure 7A). The N-terminal contains most of the active site residues, the binding site for PLP and the dimerization interface (Figure 7A). The binding site is formed by the residues Trp108 and Val185, where the pyridoxal ring of PLP is stacked, and Lys209, which binds to PLP via a Schiff base linkage (Figure 7A). Human AGXT has two polymorphic variants: the ‘major’ allele (AGT WT, present in 80% of human alleles) and the ‘minor’ allele (AGT LM, present in 20% of human alleles). AGT LM is characterized by two single residue substitutions (P11L and I340M) and a 74 bp duplication in intron 1 [143]. Most primary hyperoxaluria type 1 (PH1) mutations are found in either the major or minor allele, but rarely in both [139]. Although AGT LM alone cannot cause PH1, it exacerbates the loss-of-function effects of several common mutations [140]. The most common mutations in AGTX result in four main mechanisms: mitochondrial mistargeting, protein aggregation, catalytic defects, and synthesis defects [144,145], with mitochondrial mistargeting being the most studied, particularly involving the G170R substitution in the minor allele [144,146], where the P11L polymorphism causes incorrect protein folding, preventing homodimer formation and compromising peroxisomal import of AGT [145,147,148,149].

Despite the wealth of biochemical, functional, and structural data available for AGT, a better understanding of the reaction mechanism for the conversion of L-alanine and glyoxylate to pyruvate and glycine by AGT (Figure 7B) has been hampered by ignorance of the dynamics of AGT. Although promising treatment options for PH1 based on RNA interference technology have recently been approved by both the FDA and EMA, and others are currently being tested at the clinical level [150,151], the availability of treatment strategies based on small-molecule drugs is an unmet need. Thus, the combination of structural and dynamics data could be very useful to guide the drug discovery process, paving the way for further medicinal chemistry optimization strategies. In this regard, a recent study using hydrogen-deuterium exchange monitored by mass spectrometry to provide the first experimental analysis of the local stability and dynamics of AGT, showing that stability is heterogeneous in the native state and providing a blueprint for dynamic regions with potentially functional relevance [152]. Furthermore, this study reports that AGT possesses high plasticity, which may indicate that the mechanism used by this enzyme to process its substrates may be coupled to some kind of cooperative mechanism (or allostery) [152].

With the final goal of better understanding the individual pathways and the possible interconnection between them, time-resolved serial crystallography experiments could be implemented to provide insight into the conformational changes in the AGT native state stability between two polymorphic variants (WT and LM) and the most common PH1-causing mutations and to provide information on how the folding landscape is affected. In previous investigations, it has also been hypothesized that the use of different ligands could improve the stability of the enzyme. To achieve this aim, P. Vankova et al. [152] compared the stability and dynamics of AGT when the PLP or PMP (pyridoxamine phosphate) is bound by using hydrogen-deuterium exchange mass spectrometry; however, they were not able to observe differences between the two bound states of AGT. Understanding the structure-function relationships in AGT and its interaction with its substrate L-alanine at the atomic level will be critical to unravel AGT’s role in the glyoxylate detoxification and to identify a potential target to treat PH1. To this end, time-resolved serial crystallography experiments would pinpoint the structure of the reaction intermediates involved in the reductive and oxidative half-reactions of the ‘ping-pong’ mechanism of AGT (Figure 7B), along with the concomitant conformational changes, to obtain the first molecular movie of the reaction mechanism in AGT. Thus, knowledge of the mechanism of AGT would be the first step in the pharmaceutical design of novel agents that might enhance its overall rate of productive folding and dimerization and increase the stability of the dimer once formed.

### 7.6. Human Pyridoxine 5′-Phosphate Oxidase (PNPOx)

All living beings rely on vitamin B6 for their existence; however, only microorganisms and plants can synthesize it *de novo*. Humans, like all other mammals, obtain PLP from B6 vitamins acquired from the diet and recycled in a salvage pathway involving phosphatases, an ATP-dependent pyridoxal kinase (PLK) and a flavin mononucleotide (FMN)-dependent pyridoxine (pyridoxamine) 5′-phosphate oxidase (PNPOx). The human PNPOx (EC 1.4.3.5) is a vital enzyme involved in vitamin B6 metabolism. PNPOx converts pyridoxine 5′-phosphate (PNP) into pyridoxal 5′-phosphate (PLP), the active form of vitamin B6 essential for various biological processes. PNPOx also recycles PMP to regenerate PLP, the active cofactor. PLP is the cofactor in over 140 enzyme-catalyzed reactions in human cells, including many involved in the synthesis or degradation of amino acids or amines that serve as neurotransmitters or neuromodulators in the brain [153,154].

The interaction between PLP, the product of PNPOx, and its recipient apo-enzymes is vital for their enzymatic activity, folding, and cellular localization [155]. PLP deficiency, which is linked to various neurological and non-neurological disorders (e.g., epilepsy), is rarely due to dietary insufficiency, given the widespread presence of vitamin B6 in most diets. Malfunctions in enzymes like PLK and PNPOx, caused by inherited mutations or drug-induced inhibition, are major contributors to PLP deficiency [156,157]. Drug-induced inhibition may manifest in symptoms like unconsciousness, seizures, and tremors, while inherited mutations are associated with severe pathologies including neonatal epileptic encephalopathy, autism, schizophrenia, and neurodegenerative diseases like Parkinson’s and Alzheimer’s, among others. On the other hand, excess vitamin B6 can lead to toxicity [158,159]. The pool of free PLP in vivo is maintained at a very low level in the body, presumably to prevent toxic buildup. PLP’s highly reactive aldehyde group at the 4′ position makes it useful for protein labeling but also contributes to its potential toxicity. Elevated B6 levels may result from environmental factors or genetic defects. Vitamin B6 toxicity can induce sensory and motor neuropathies, causing numbness in the hands and feet, which are usually reversible upon discontinuation of supplementation [160]. Yet, substantial amounts of this cofactor are essential to saturate the numerous PLP-dependent enzymes and meet cellular demands. Understanding the mechanisms governing PLP homeostasis regulation and its delivery to apoenzymes requiring it as a cofactor remains an important, yet unresolved, goal.

Structurally speaking, PNPOx is a symmetric homodimeric enzyme with two FMN-binding sites. Each monomer is made up of a two-domain α/β-barrel fold (Figure 5B). The binding site of FMN is in a deep cleft formed by the two subunits and makes extensive hydrogen bond interactions with highly conserved residues from both subunits [161] (Figure 5B). The crystal structure of PNPOx in its unliganded form shows an open active site [161]; however, the crystal structure of PNPOx in complex with PLP shows previously unobserved N-terminal residues that fold over the active site to completely close it [162]. In another crystal structure of PNPOx in complex with PLP, a second PLP binds to PNPOx approximately 11 Å away from the active site, suggesting this may correspond to the tight PLP-binding site [163]. This second PLP molecule exhibits two conformations, with differing occupancies. The conformer with higher occupancy resides in a cavity formed by flexible loops, sandwiched between Phe177 and Lys145. Adjacent to Phe177 is Asn84, which forms a short hydrogen bond with the pyridine nitrogen of PLP. The presence of Trp178, Phe177, Arg133, and Asn84 narrows the putative tunnel between the two PLP binding sites. Although the tunnel appears small for PLP passage, the surrounding protein structures are flexible, suggesting that the channel could expand. Understanding the role of the non-catalytic site in channeling sequestered PLP to other enzymes is an important area for further investigation. The binding of PLP to this site may regulate the free PLP concentration *in vivo*. Thus, continuing research in the field of PLP transfer and channeling to PLP-dependent enzymes is key because we have limited molecular knowledge of these processes.

Despite all the structural information available, the mechanisms by which PNPOx converts PNP into PLP and the transfer and channeling of the PLP to PLP-dependent enzymes remain elusive. Thus, a deeper understanding of such mechanisms will surely lead to insights into the regulation of metabolic pathways and the mechanisms underlying diseases associated with PLP-dependent enzyme dysfunction. To this end, TR-SX holds great promise for elucidating the reaction mechanism of PNPOx with PNP and the subsequent transfer and channeling to PLP-dependent enzymes by the following:

1. Capturing reaction intermediates: Monitoring the structural changes of PNPOx and its active site residues during the catalytic cycle will allow us to elucidate the catalytic mechanism of PNPOx, including the proton transfer steps.
2. Investigating transfer and channeling mechanism: By capturing the structural dynamics of PNPOx in complex with PLP and potential protein partners involved in PLP transfer, it will be possible to gain more insights into the mechanisms underlying substrate channeling and enzyme regulation.
3. Identifying allosteric regulation: Understanding the allosteric regulation of PNPOx will be critical for deciphering its physiological roles and developing allosteric modulators as potential therapeutic agents.

Overall, TR-SX offers a powerful tool for dissecting the complex reaction mechanism of PNPOx with PNP and the subsequent transfer and channeling to PLP-dependent enzymes by providing unprecedented atomic-level insights into the enzyme dynamics and substrate interactions. Uncovering the intricate details of such processes will help to identify potential targets for therapeutic interventions and develop novel treatments for vitamin B6 deficiency disorders, epilepsy, and even certain types of cancer. The precise coordination required for PLP delivery to specific enzymes highlights the complexity of cellular regulation and the interconnectedness of metabolic pathways.

## 8. Conclusions and Outlook

This review underscores the profound advancements brought about by XFELs in the field of structural biology, particularly in understanding enzyme dynamics within metabolic disorders. Traditional X-ray crystallography, while pivotal in providing high-resolution static images of enzymes, has notable limitations, such as the requirement for suitable protein crystals and the inability to capture protein dynamics effectively. XFELs, with their ultra-short and intense X-ray pulses, have overcome these limitations, allowing researchers to obtain high-resolution structures of radiation-sensitive and difficult-to-crystallize proteins at room temperature, and to observe enzyme dynamics through time-resolved serial femtosecond crystallography. The insights gained from these dynamic studies are critical for elucidating the molecular mechanisms underlying metabolic diseases. By capturing the transient states and conformational changes of enzymes during catalysis, researchers can better understand the specific alterations in enzyme function that contribute to disease states. This knowledge is essential for the development of targeted therapies aimed at correcting or mitigating these dysfunctions. Furthermore, with this review, we aimed to underline the significance of microcrystals in studying enzyme dynamics. Microcrystals facilitate the rapid diffusion of substrates, making them ideal for time-resolved studies. The advent of techniques such as mix-and-inject serial crystallography has further expanded the capability to investigate enzyme reactions in real time, providing a more comprehensive understanding of enzyme mechanisms and interactions.

To support this contention, we reported some examples to demonstrate the potentiality of the integration of XFELs and SX. (1) NQO1 is crucial for cardiovascular health, reducing oxidative stress and modulating inflammation. TR-SFX experiments have provided insights into its catalytic mechanism, showing dynamic changes during redox reactions. Recent advancements in crystallography have revealed new aspects of NQO1’s flexibility and intermediates. (2) GAC is essential in glutaminolysis, supporting cancer cell metabolism. Inhibiting GAC can disrupt cancer cell survival. TR-SX provides insights into GAC’s catalytic cycle and inhibitor binding, aiding in the design of cancer therapies targeting glutamine metabolism. (3) GPCRs are vital for cell signaling, but low expression and crystallization challenges hinder X-ray crystallography. SFX, XFELs, and cryo-EM have revolutionized GPCR structural biology, revealing novel structures and complex interactions. Integrative approaches combining SFX, cryo-EM, and NMR are key to understanding GPCR signaling and designing effective therapies. (4) CYP2D6 is critical in drug metabolism. Understanding its structure and dynamics is vital for predicting drug interactions and optimizing therapies. TR-SFX has provided snapshots of CYP2D6’s catalytic cycle, enhancing understanding of its mechanisms and supporting the development of personalized medicines. (5) AGT detoxifies glyoxylate to prevent oxalate buildup in primary hyperoxaluria type I (PH1). Structural studies show AGT to be a homodimeric enzyme with critical active site and dimerization interfaces. TR-SX can elucidate AGT’s mechanisms, aiding in drug discovery for PH1. (6) PNPOx converts PNP to PLP in vitamin B6 metabolism. Dysfunctions in PNPOx can cause PLP deficiency, leading to neurological disorders, while excess PLP causes toxicity. TR-SX can offer insights into PNPOx’s catalytic cycle and regulation, guiding therapies for vitamin B6 deficiency.

In conclusion, the integration of advanced structural techniques like TR-SX with traditional methods offers a comprehensive understanding of enzyme function and dynamics. Through the application of TR-SX, researchers will be able to elucidate the kinetic properties, substrate specificity, and regulatory mechanisms of metabolism-related enzymes. This knowledge will be crucial in understanding the role of these enzymes in maintaining cellular homeostasis and identifying potential targets for therapeutic intervention. The insights gained from TR-SX studies can open up new avenues for drug discovery. By identifying key enzymes involved in metabolic pathways associated with diseases, researchers can now focus on developing targeted therapies that modulate enzyme activity. TR-SX can be used to screen large chemical libraries for potential drug candidates and to optimize lead compounds for improved potency and selectivity. As the field of metabolism research continues to evolve, the application of TR-SX will undoubtedly play an increasingly important role. Advances in instrumentation and data analysis techniques will further enhance the sensitivity and throughput of TR-SX assays, enabling the study of more complex enzyme systems and the discovery of novel therapeutic targets. TR-SX will remain an essential tool for advancing our understanding of cellular metabolism and its implications for human health.

## Data Availability

No new data were created or analyzed in this study. Data sharing is not applicable to this article.
